# 恶性血液病患者发生铜绿假单胞菌感染的临床特征及预后分析

**DOI:** 10.3760/cma.j.cn121090-20240824-00319

**Published:** 2024-11

**Authors:** 海枝 高, 璐婷 罗, 丽华 芦, 晓云 郑, 婷 杨, 建达 胡

**Affiliations:** 1 福建医科大学附属协和医院血液科，福州 350001 Fujian Medical University Union Hospital, Fuzhou 350001, China; 2 福建医科大学附属南平第一医院血液科，南平 353000 Nanping First Hospital of Fujian Medical University, Nanping 353000, China; 3 福建医科大学附属第二医院血液科，泉州 362000 The Second Hospital of Fujian Medical University, Quanzhou 362000, China; 4 福建医科大学附属第一医院滨海校区、国家区域医疗中心血液科，福州 350200 National Regional Medical Centre, The First Affiliated Hospital of Fujian Medical University, Binhai Campus, Fuzhou 350200, China; 5 福建医科大学精准医学研究院，福州 350004 Institute of Precision Medicine, Fujian Medical University, Fuzhou 350004, China; 6 福建医科大学附属第一医院血液科，福州 350004 The First Hospital of Fujian Medical University, Fuzhou 350004, China

**Keywords:** 铜绿假单胞菌感染, 碳青霉烯敏感铜绿假单胞菌, 碳青霉烯耐药铜绿假单胞菌, 血流感染, Pseudomonas aeruginosa infection, Carbapenem-sensitive Pseudomonas aeruginosa, Carbapenem-resistant Pseudomonas aeruginosa, Bloodstream infection

## Abstract

**目的:**

探讨恶性血液病患者发生铜绿假单胞菌感染的临床特征及预后影响因素。

**方法:**

回顾性分析2019年1月1日至2021年12月31日期间在福建医科大学附属协和医院血液科住院的197例合并铜绿假单胞菌感染的恶性血液病患者的临床资料。根据对碳青霉烯类药物的敏感性分为敏感组（CSPA感染组）和耐药组（CRPA感染组），比较两组患者临床特征的差异，分析容易发生CRPA感染的危险因素及预后情况。

**结果:**

Logistic回归多因素分析显示住院天数>50 d（*P*＝0.010，*OR*＝3.581，95％*CI* 1.356～9.457）、抗生素暴露史（*P*＝0.008，*OR*＝4.394，95％*CI* 1.358～6.238）、感染前化疗次数>2次（*P*＝0.006，*OR*＝2.911，95％*CI* 1.358～6.238）为发生CRPA感染的独立危险因素。CSPA感染组和CRPA感染组30 d全因死亡率分别为12.8％（18/140）、28.1％（16/57）（*P*＝0.010）。Logistic回归多因素分析显示血流感染（*P*＝0.039，*OR*＝5.286，95％*CI* 1.091～25.621）是恶性血液病伴CRPA感染死亡的独立危险因素。

**结论:**

住院天数>50 d、抗生素暴露史、感染前化疗次数>2次为恶性血液病患者发生CRPA感染的独立风险因素。血液病患者CRPA感染后死亡率高，血流感染为其主要死亡原因。

## Abstract (en)

**Objective:**

This study aimed to investigate the clinical features and prognosis of Pseudomonas aeruginosa infection in patients with hematologic malignancies.

**Methods:**

This study retrospectively analyzed the clinical data of 197 patients with hematologic malignancies complicated with P. aeruginosa infection who were hospitalized in the Department of Hematology from January 01, 2019, to December 31, 2021. Patients were categorized into a susceptible group（CSPA infection group）and a drug-resistant group（CRPA infection group）based on their sensitivity to carbapenems, comparing the differences in clinical features between the two groups, and analyzing the risk factors and prognosis of CRPA infection.

**Results:**

Logistic regression analysis revealed that hospitalization days of >50 days（*P*＝0.010, *OR*＝3.581, 95％ *CI* 1.356–9.457）, history of antibiotic exposure（*P*＝0.008, *OR*＝4.394, 95％ *CI* 1.358–6.238）, more than two courses of chemotherapy before infection（*P*＝0.006, *OR*＝2.911, 95％ *CI* 1.358–6.238）were independent risk factors for developing CRPA. The mortality rates were 12.8％（18/140）and 28.1％（16/57）in patients with CRPA and CSPA, respectively（*P*＝0.010）. Logistic regression analysis revealed that bloodstream infection（BSI）（*P*＝0.039, *OR*＝5.286, 95％ *CI* 1.091–25.621）was an independent risk factor for hematologic malignancies and death from CRPA infection.

**Conclusion:**

Hospitalization for >50 days, history of antibiotic exposure, and >2 courses of chemotherapy before infection were independent risk factors for CRPA infection. Hematologic malignancies with CRPA infection had a high mortality rate, of which BSI was an independent risk factor for 30-day mortality from hematologic malignancies with CRPA infection.

国内多中心研究结果显示，粒细胞缺乏（粒缺）伴发热最常见的病原菌为革兰阴性菌，其中铜绿假单胞菌排革兰阴性菌第3位[1]。2022年中国细菌耐药监测网（CHINET）数据显示，临床常见菌株以革兰阴性菌为主，其中铜绿假单胞菌27 257株（占革兰阴性菌11.3％），占第3位。既往我科共分离出的181株碳青霉烯类耐药革兰阴性菌（CRO），耐碳青霉烯类非发酵菌和耐碳青霉烯类肠杆菌分别占52.5％（95/181）和46.9％（85/181）；菌株以铜绿假单胞菌为主37.0％（67/181），其次为肺炎克雷伯菌32.6％（59/181）[2]。铜绿假单胞菌是导致血流感染（bloodstream infection, BSI）的常见病原菌[3]，碳青霉烯类耐药铜绿假单胞菌（CRPA）BSI 30 d全因死亡率可达到39％[4]。国内多个研究显示，与碳青霉烯类敏感铜绿假单胞菌（CSPA）相比，CRPA引起的感染与更高的死亡率以及住院费用有关[5]–[9]。鉴于铜绿假单胞菌常造成严重感染，尤其是恶性血液病患者出现碳青霉烯类药物耐药，抗感染治疗难度大，死亡率高，临床医师抗感染治疗面临极大挑战。WHO在2017年已将CRPA危险级别确定为“紧要优先”（critical-priority）[10]。本文通过回顾性分析2019年1月1日至2021年12月31日在福建医科大学附属协和医院血液科住院的恶性血液病患者发生铜绿假单胞菌感染的临床特征，分析CRPA感染的预后及其危险因素，现报道如下。

## 病例与方法

一、病例资料

回顾性收集2019年1月1日至2021年12月31日期间福建医科大学附属协和医院血液科住院治疗的恶性血液病伴铜绿假单胞菌感染患者的临床资料。所有患者诊断符合恶性血液病诊断标准，标本类型包括血液、痰液、咽拭子、肛周拭子、中段尿液等，根据《医院感染诊断标准（试行）》[11]，确定致病菌为铜绿假单胞菌。如果同一患者有2次或2次以上细菌培养显示铜绿假单胞菌阳性，本研究只纳入首次感染时的资料。排除病例资料不完整患者。

二、病原菌分离、鉴定及药敏分析

病原菌分离及鉴定严格按《全国临床检验操作规程》的相关规定进行，采用法国生物梅里埃公司的全自动微生物分析系统（VITEK2 Compact）进行细菌鉴定和药敏试验，药敏试验结果参考2018年美国临床实验室标准化协会（CLSI）[12]标准判读。对所有检测的碳青霉烯类药物如美罗培南和亚胺培南同时耐药，最小抑菌浓度（MIC）≥8 mg/L定义为碳青霉烯类耐药。

三、研究方法

收集患者一般临床特征及相关实验室检查资料。根据对碳青霉烯类药物的敏感性将患者分为CSPA感染组和CRPA感染组，分析发生CRPA感染的危险因素及CRPA感染亚组30 d全因死亡的危险因素。

四、相关定义

抗菌药物暴露：发病前1个月内全身使用抗菌药物>3 d。粒缺定义为中性粒细胞绝对计数<0.5×10^9^/L。

五、随访

通过医院住院病历管理系统、病案室文档、电话回访等方式进行随访，主要随访指标为铜绿假单胞菌阳性标本留取后30 d的生存情况，随访截止时间为2022年1月31日。

六、统计学处理

采用SPSS 25.0统计软件进行统计分析。临床资料中连续性变量若为正态分布，采用均数±标准差来描述连续性变量的分布，检验方法为*t*检验；若为偏态分布，采用中位数和四分位距（Interquartile range，*IQR*）来描述变量的分布，检验方法为非参数检验。分类资料的描述采用例数和相应分类百分比表示，组间比较采用卡方检验或Fisher精确概率法。将单因素分析*P*<0.05的变量纳入多因素分析模型，计算比值比（*OR*）和95％置信区间（*CI*）。所有检验，以*P*<0.05为差异有统计学意义。

## 结果

一、一般临床特征

共纳入恶性血液病伴铜绿假单胞菌感染患者197例，其中男性116例（58.9％），中位年龄49（35，61）岁。疾病类型：急性髓系白血病88例（44.7％），急性淋巴细胞白血病33例（16.8％），淋巴瘤41例（20.8％），骨髓增生异常综合征14例（7.1％），多发性骨髓瘤6例（3.0％），其他15例（7.6％）。20例患者合并糖尿病（10.2％）。既往1年内行造血干细胞移植39例（19.8％），感染前接受糖皮质激素治疗者84例（42.6％），合并粒缺125例（63.5％），留置中心静脉置管173例（87.8％）。送检标本类型主要为血液（48株，23.4％）、痰（66株，33.5％）、口咽拭子（45株，22.8％）。共检出140株CSPA菌株，57株CRPA菌株，92％的CSPA菌株分别从痰（31.4％）、血液（26.4％）、口咽拭子（21.4％）、肛周拭子（6.4％）和创面分泌物（6.4％）中检出。91.2％的CRPA菌株分别从痰（38.6％）、口咽拭子（26.3％）、血液（19.3％）和肛周拭子（7.0％）中检出。详见表1。

**表1:** 197例恶性血液病患者伴铜绿假单胞菌感染临床特征

临床特征	总体（197例）	CSPA感染组（140例）	CRPA感染组（57例）	*P*值
年龄［岁，*M*（*IQR*）］	49（35, 61）	51（36, 63）	44（33, 58）	0.037
男性［例（％）］	116（58.9）	80（57.1）	36（63.1）	0.437
疾病类型［例（％）］				
AML	88（44.7）	58（41.4）	30（52.6）	0.332
ALL	33（16.8）	21（15.0）	12（21.1）	
淋巴瘤	41（20.8）	34（24.3）	7（12.3）	
MDS	14（7.1）	11（7.9）	3（5.3）	
MM	6（3.0）	5（3.6）	1（1.8）	
其他	15（7.6）	11（7.9）	4（7.0）	
合并糖尿病［例（％）］	20（10.2）	16（11.4）	4（7.0）	0.353
1年内行造血干细胞移植［例（％）］	39（19.8）	17（12.1）	22（38.6）	0.001
感染前糖皮质激素治疗［例（％）］	84（42.6）	55（39.3）	29（50.9）	0.136
粒细胞缺乏［例（％）］	125（63.5）	90（64.3）	35（61.4）	0.703
感染前化疗次数［*M*（*IQR*）］	2（1, 5）	2（1, 4）	3（1, 5）	0.005
中心静脉置管［例（％）］	173（87.8）	122（87.1）	51（89.4）	0.650
住院天数>50 d	26（13.9）	11（7.8）	15（26.3）	0.001
白蛋白<30 g/L［例（％）］	83（42.1）	57（40.7）	26（45.6）	0.528
HGB［g/L，*M*（*IQR*）］	62（52.0, 83.0）	63.0（52.0, 84.0）	60.0（53.0, 76.5）	0.506
PLT［×10^9^/L，*M*（*IQR*）］	24（9.0, 65.5）	24.5（9.0, 91.5）	19.0（8.0, 47.0）	0.129
ANC［×10^9^/L，*M*（*IQR*）］	0.24（0.03, 2.35）	0.24（0.03, 2.02）	0.44（0.04, 2.65）	0.742
感染部位［例（％）］				0.001
肺部	66（33.5）	44（31.4）	22（38.6）	
血流	48（24.3）	37（26.4）	11（19.3）	
口腔	45（22.8）	30（21.4）	15（26.3）	
肛周	13（6.6）	9（6.4）	4（7.0）	
皮肤软组织	12（6.1）	9（6.4）	3（5.3）	
泌尿系统	9（4.6）	7（5.0）	2（3.5）	
眼部	3（1.5）	3（2.1）	0（0）	
阴道	1（0.5）	1（0.7）	0（0）	

**注** CSPA：碳青霉烯类敏感铜绿假单胞菌；CRPA：碳青霉烯类耐药铜绿假单胞菌；AML：急性髓系白血病；ALL：急性淋巴细胞白血病；MDS：骨髓增生异常综合征；MM：多发性骨髓瘤

二、恶性血液病患者发生CRPA感染的相关因素分析

197例恶性血液病患者发生CRPA感染的单因素分析显示，感染前糖皮质激素治疗、1年内行造血干细胞移植、住院天数>50 d、抗生素暴露史、感染前化疗次数>2次是发生CRPA感染的影响因素（均*P*<0.05）。而中心静脉置管、导尿、粒缺持续时间≤14 d在单因素分析中无统计学意义（均*P*>0.05）（表2）。将单因素分析中*P*<0.05的危险因素纳入多因素分析，结果显示住院天数>50 d、抗生素暴露史、感染前化疗次数>2次为发生CRPA感染的独立危险因素（表3）。

**表2:** 影响197例恶性血液病患者发生CRPA感染的单因素分析［例（％）］

影响因素	CSPA感染组（140例）	CRPA感染组（57例）	*χ*^2^值	*P*值
感染前糖皮质激素治疗	71（50.7）	41（71.9）	7.433	0.006
1年内行造血干细胞移植	17（12.1）	22（38.6）	17.853	0.001
住院天数>50 d	11（7.9）	15（26.3）	12.048	0.001
年龄≤50岁	69（42.3）	38（66.7）	5.171	0.075
抗生素暴露史	104（74.3）	52（91.2）	7.005	0.008
感染前化疗次数>2次	57（40.77）	37（64.9）	9.507	0.002
中心静脉置管	122（87.1）	51（89.5）	0.206	0.650
导尿	14（10.0）	9（15.8）	1.317	0.251
粒缺持续时间≤14 d	102（72.9）	38（66.7）	0.755	0.385

**注** CSPA：碳青霉烯类敏感铜绿假单胞菌；CRPA：碳青霉烯类耐药铜绿假单胞菌；粒缺：粒细胞缺乏

**表3:** 影响197例恶性血液病患者发生碳青霉烯类耐药铜绿假单胞菌感染的多因素分析［例（％）］

影响因素	*OR*	95％*CI*	*P*值
感染前糖皮质激素治疗	1.926	0.918～4.044	0.083
1年内造血干细胞移植	2.064	0.864～4.931	0.103
住院天数>50 d	3.581	1.356～9.457	0.010
感染前化疗次数>2次	2.911	1.358～6.238	0.006
抗生素暴露史	4.394	1.466～13.168	0.008

三、恶性血液病伴铜绿假单胞菌感染死亡率分析

197例恶性血液病合并铜绿假单胞菌感染患者30 d内死亡34例（17.3％），其中CSPA感染组140例患者死亡18例（12.8％），而CRPA感染组57例患者死亡16例（28.1％），差异有统计学意义（*P*＝0.010）。

四、恶性血液病伴CRPA感染死亡相关因素分析

在57例恶性血液病CRPA感染患者中进行30 d死亡影响因素分析，单因素分析结果显示BSI、白蛋白<30 g/L、PLT、ANC均与患者的预后不良相关（均*P*<0.05）（表4）。将单因素分析中*P*<0.05的因素纳入多因素分析，结果显示血流感染是恶性血液病伴CRPA感染死亡的独立危险因素（表5）。

**表4:** 影响57例恶性血液病患者碳青霉烯类耐药铜绿假单胞菌感染30 d死亡的单因素分析

影响因素	生存组（41例）	死亡组（16例）	统计量	*P*值
年龄［岁，*M*（*IQR*）］	39（32.5，57.5）	38（31，46.5）	−0.115	0.908
男性［例（％）］	26（63.4）	10（62.5）	0.004	0.949
疾病类型［例（％）］				0.722
ALL	10（24.4）	3（18.7）		
AML	22（53.7）	8（50.0）		
MDS	1（2.4）	2（12.5）		
淋巴瘤	5（12.2）	2（12.5）		
其他	3（7.3）	1（6.3）		
感染前化疗疗程>2次［例（％）］	30（73.2）	8（50.0）	2.780	0.095
1年内行造血干细胞移植［例（％）］	17（41.4）	5（31.3）	0.507	0.477
血流感染［例（％）］	4（9.7）	7（43.8）		0.002
感染前糖皮质激素治疗［例（％）］	31（75.6）	10（62.5）	0.980	0.322
中心静脉置管［例（％）］	35（85.4）	16（100.0）		0.170
抗生素暴露史［例（％）］	36（87.8）	16（100.0）		0.308
粒缺［例（％）］	22（53.7）	13（81.3）	3.697	0.055
住院天数>50 d［例（％）］	9（22.0）	6（37.5）		0.317
白蛋白<30 g/L［例（％）］	15（36.6）	11（68.8）	4.800	0.028
HGB［g/L，*M*（*IQR*）］	66.5（55.2，88.0）	57.5（49.5，72.8）	−1.093	0.274
PLT［×10^9^/L，*M*（*IQR*）］	25.5（13.2，50.5）	13.5（3.8，48.8）	−2.133	0.033
ANC［×10^9^/L，*M*（*IQR*）］	0.93（0.05，3.27）	0.13（0.05，0.64）	−2.143	0.032

**注** ALL：急性淋巴细胞白血病；AML：急性髓系白血病；MDS：骨髓增生异常综合征；粒缺：粒细胞缺乏

**表5:** 影响57例恶性血液病患者碳青霉烯类耐药铜绿假单胞菌感染30 d死亡的多因素分析

影响因素	*OR*	95％*CI*	*P*值
血流感染	5.286	1.091～25.621	0.039
白蛋白<30 g/L	2.904	0.746～11.314	0.124
ANC（×10^9^/L）	0.873	0.567～1.342	0.535
PLT（×10^9^/L）	0.993	0.972～1.014	0.513

## 讨论

本组恶性血液病患者发生铜绿假单胞菌感染的主要部位为呼吸道、血液、口腔，与文献[13]研究一致；而部分研究显示铜绿假单胞菌感染常见部位为呼吸道，其次为尿液、脓液、血液等，其中BSI占比较小[14]，与本研究不一致，这可能与本次研究以恶性血液病为基础，因恶性血液病反复化疗、机体抵抗力差、免疫功能低下，多数患者长期留置中心静脉导管等因素相关。

闫晨华等[1]的研究提示粒缺伴发热是血液科患者常见的并发症，本次研究纳入197例恶性血液病伴铜绿假单胞菌感染人群中粒缺人数占比高达63.5％，与上述研究一致。而CSPA感染组及CRPA感染组中粒缺患者占比相近（*P*＝0.703），无法说明粒缺可增加CRPA感染风险。孟庆彩等[15]研究表明粒缺程度、持续时间与感染的发生率及严重程度相关，粒缺持续时间愈长则感染的发生率就越高，病情亦越重。故粒缺患者应重视感染的预防，粒缺伴发热需及时进行危险分层、耐药危险因素评估，根据本区域、本院及本科室感染的流行病学覆盖耐药菌选择合适抗生素，尽早抗感染治疗，必要时重拳出击，避免感染加重，降低死亡率[16]。

本研究针对197例恶性血液病发生CRPA感染单因素分析显示CRPA感染组感染前糖皮质激素治疗、1年内行造血干细胞移植、感染前化疗次数>2次、既往抗生素暴露史的比例均高于CSPA感染组。这与恶性血液病治疗的特殊性相关，原发病治疗过程中行造血干细胞移植、多疗程化疗及使用激素的情况下，使机体达深度免疫抑制，可能导致上皮细胞损伤（黏膜炎），破坏抵抗内源性菌群入侵的屏障，使感染极易发生[17]。暴露于抗生素后可引起耐药（甚至是多重耐药），其原因可能是抗生素灭活酶的产生、细菌外膜的通透性低、各种外排泵的过表达以及外膜蛋白的丢失[18]。有研究表明长时间使用抗生素与多重耐药铜绿假单胞菌感染（MDR-PA）相关[19]，MDR会增加PA BSI患者死亡风险，既往抗生素暴露会增加MDR-PA BSI发生率[20]。也有研究表明MDR-PA引起的BSI是30 d死亡的独立危险因素[8]。本文多因素分析中结果显示既往抗生素暴露史为发生CRPA的独立危险因素。综上提示抗生素暴露对MDR-PA及CRPA的发生很重要，临床工作中需根据当地抗生素敏感性及临床特点合理使用抗生素，避免抗生素长期暴露导致细菌耐药性发生、降低死亡率。同时多因素分析中显示住院天数>50 d、感染前化疗次数>2次为发生CRPA的独立危险因素，与文献结果基本一致[5],[13],[21]。住院时间的延长，增加了定植菌转移以及交叉感染的机会，缩短住院天数可减少CRPA感染发生率。

本组患者30 d内全因死亡率为17.3％。CSPA感染组和CRPA感染组30 d全因死亡率分别为12.8％、28.1％（*P*＝0.010），提示CRPA感染预后不良，这与国内相关数据一致[9]。Bergas等[22]研究显示严重的中性粒细胞减少症（ANC<0.1×10^9^/L）是恶性血液病患者铜绿假单胞菌BSI 30 d死亡的独立危险因素（*P*＝0.001）。也有研究表明PA BSI后粒缺持续时间>7 d患者的30 d死亡率显著增加（34.5％对4.4％，*P*<0.001）[8]。这与本次CRPA感染亚组死亡单因素分析中提示较低的ANC为患者预后不良因素一致，这可能与长期粒缺可影响抗菌药物对铜绿假单胞菌清除能力相关，故缩短粒缺持续时间尤为重要。但是多因素分析仅提示BSI为恶性血液病伴CRPA感染死亡的独立危险因素。故避免CRPA BSI是降低死亡率的关键。彭苏琴[23]的研究证明既往1个月内抗生素暴露为发生CRPA BSI的独立危险因素。Shi等[24]的回顾性研究显示，BSI发病前90 d内暴露于碳青霉烯类药物（*OR*＝1.987，95％*CI* 1.052～3.753，*P*＝0.034）及抗真菌药物（*OR*＝2.258，95％*CI* 1.166～4.371，*P*＝0.016）为发生CRPA BSI的独立危险因素。总之，既往抗生素暴露会导致细菌耐药性风险增加，是发生CRPA BSI的危险因素，而CRPA BSI感染死亡风险高，治疗难度大，故早期、合理使用抗菌药物尤为关键。

综上所述，恶性血液病发生CRPA感染的死亡率较高，BSI为其主要原因，通过对恶性血液病患者发生CRPA感染危险因素的分析，希望可以指导临床医务人员在工作中合理及时使用抗菌药物，缩短住院时长，减少CRPA感染发生，降低恶性血液病发生CRPA感染死亡率。

## References

[1] 闫 晨华, 徐 婷, 郑 晓云 (2016). 中国血液病患者中性粒细胞缺乏伴发热的多中心、前瞻性流行病学研究[J]. 中华血液学杂志.

[2] 陈 少桢, 许 晶晶, 肖 婷婷 (2021). 血液科碳青霉烯耐药革兰阴性菌感染的临床特征及预后危险因素分析[J]. 中华血液学杂志.

[3] 王 盼, 别 甜敏, 金 文平 (2023). 2012-2021年间血流感染病原菌分布及耐药性分析[J]. 中国抗生素杂志.

[4] Yuan Q, Guo L, Li B (2023). Risk factors and outcomes of inpatients with carbapenem-resistant Pseudomonas aeruginosa bloodstream infections in China: a 9-year trend and multicenter cohort study[J]. Front Microbiol.

[5] 施 清怡 (2019). 碳青霉烯类不敏感铜绿假单胞菌血流感染危险因素及预后分析[D]. 浙江大学.

[6] 沈 英英, 赵 越超, 王 博 (2023). 血液病患者定植或感染碳青霉烯类耐药的铜绿假单胞菌的临床特征及生存分析[J]. 中国实验血液学杂志.

[7] Chen Z, Xu Z, Wu H (2019). The impact of carbapenem-resistant Pseudomonas aeruginosa on clinical and economic outcomes in a Chinese tertiary care hospital: A propensity score-matched analysis[J]. Am J Infect Control.

[8] Zhen S, Zhao Y, Chen Z (2023). Assessment of mortality-related risk factors and effective antimicrobial regimens for treatment of bloodstream infections caused by carbapenem-resistant Pseudomonas aeruginosa in patients with hematological diseases[J]. Front Cell Infect Microbiol.

[9] 李 霖, 黄 文治, 乔 甫 (2023). 耐碳青霉烯类铜绿假单胞菌血流感染患者的预后及其影响因素研究[J]. 华西医学.

[10] Tacconelli E, Carrara E, Savoldi A (2018). Discovery, research, and development of new antibiotics: the WHO priority list of antibiotic-resistant bacteria and tuberculosis[J]. Lancet Infect Dis.

[11] 中华人民共和国卫生部 (2001). 医院感染诊断标准(试行)[J]. 中华医学杂志.

[12] Clinical And Laboratory Standards Institutes (2018). Performance standards for antimicrobial susceptibility testing[S]. M100S ed.

[13] 骆 宜茗, 刘 庭波, 谢 泗停 (2015). 成人急性白血病患者住院化疗感染的临床特征及影响因素研究[J]. 中华血液学杂志.

[14] 赵 越, 黄 雅轩, 阙 熳芳 (2023). 耐碳青霉烯类铜绿假单胞菌感染的临床特征及相关危险因素研究[J]. 中国实验诊断学.

[15] 孟 庆彩, 王 卫红, 陈 爱地 (2023). 2014—2020年血液病伴中性粒细胞减少患者血流感染病原菌耐药性及预后因素分析[J]. 临床军医杂志.

[16] 中华医学会血液学分会, 中国医师协会血液科医师分会 (2020). 中国中性粒细胞缺乏伴发热患者抗菌药物临床应用指南(2020年版)[J]. 中华血液学杂志.

[17] Misch EA, Andes DR (2019). Bacterial Infections in the Stem Cell Transplant Recipient and Hematologic Malignancy Patient[J]. Infect Dis Clin North Am.

[18] Codjoe FS, Donkor ES (2017). Carbapenem Resistance: A Review[J]. Med Sci (Basel).

[19] Zhao Y, Lin Q, Zhang T (2023). Pseudomonas aeruginosa bloodstream infection in patients with hematological diseases: Clinical outcomes and prediction model of multidrug-resistant infections[J]. J Infect.

[20] Zhao Y, Lin Q, Liu L (2020). Risk Factors and Outcomes of Antibiotic-resistant Pseudomonas aeruginosa Bloodstream Infection in Adult Patients With Acute Leukemia[J]. Clin Infect Dis.

[21] Lee CH, Su TY, Ye JJ (2017). Risk factors and clinical significance of bacteremia caused by Pseudomonas aeruginosa resistant only to carbapenems[J]. J Microbiol Immunol Infect.

[22] Bergas A, Albasanz-Puig A, Fernández-Cruz A (2022). Real-Life Use of Ceftolozane/Tazobactam for the Treatment of Bloodstream Infection Due to Pseudomonas aeruginosa in Neutropenic Hematologic Patients: a Matched Control Study (ZENITH Study)[J]. Microbiol Spectr.

[23] 彭 苏琴 (2023). 铜绿假单胞菌血流感染患者预后及危险因素分析[D]. 南昌大学.

[24] Shi Q, Huang C, Xiao T (2019). A retrospective analysis of Pseudomonas aeruginosa bloodstream infections: prevalence, risk factors, and outcome in carbapenem-susceptible and -non-susceptible infections[J]. Antimicrob Resist Infect Control.

